# Downregulation of HULC Induces Ferroptosis in Hepatocellular Carcinoma via Targeting of the miR-3200-5p/ATF4 Axis

**DOI:** 10.1155/2022/9613095

**Published:** 2022-05-16

**Authors:** Lulu Guan, Feifei Wang, Mengjiao Wang, Songfeng Han, Zhaohai Cui, Shoumin Xi, Haixu Xu, Shipeng Li

**Affiliations:** ^1^Medical Research Center, Henan International Joint Laboratory of Thrombosis and Hemostasis, School of Basic Medicine, Henan University of Science and Technology, Luoyang 471000, China; ^2^Department of Geriatric Medicine, Jiaozuo People's Hospital, Xinxiang Medical University, Jiaozuo 454000, China; ^3^Department of Immunology, Key Laboratory of Immune Microenvironment and Disease of the Educational Ministry of China, Tianjin Key Laboratory of Cellular and Molecular Immunology, School of Basic Medical Sciences, Tianjin Medical University, 300070 Tianjin, China; ^4^Liver Transplantation Center, Beijing Friendship Hospital, Capital Medical University, Beijing 100050, China; ^5^Department of General Surgery, Jiaozuo People's Hospital, Xinxiang Medical University, Jiaozuo 454000, China

## Abstract

Hepatocellular carcinoma is a malignant tumor that poses a serious threat to human health. Ferroptosis, which represents an identified type of regulated iron-dependent cell death, may play an important role in hepatocellular carcinoma. However, it is unclear as to whether ferroptosis is involved with the mechanisms of lncRNA HULC in liver cancer cells. Here, we show that knockdown of HULC increases ferroptosis and oxidative stress in liver cancer cells. We also found changes in some related miRNAs in cells treated with HULC siRNA. Differential miRNA expression levels were determined with the use of high-throughput sequencing and prediction target genes identified using bioinformatics analysis. HULC was found to function as a ceRNA of miR-3200-5p, and miR-3200-5p regulates ferroptosis by targeting ATF4, resulting in the inhibition of proliferation and metastasis within HCC cells. In summary, these findings illuminate some of the molecular mechanisms through which downregulation of HULC induces liver cancer cell ferroptosis by targeting the miR-3200-5p/ATF4 axis to modulate the development of hepatocellular carcinoma.

## 1. Introduction

Hepatocellular carcinoma (HCC) represents a condition that seriously compromises human health [1]. It is the most common cause of cancer mortality worldwide [2, 3]. Molecular studies have been directed toward identifying the mechanisms of HCC cells and, in this way, may provide new targets for reducing HCC [4]. A number of cellular mechanisms related to HCC, including cell proliferation [5], apoptosis [6], and autophagy [7], have been investigated. An additional mechanism is that of ferroptosis, a recently identified type of regulated iron-dependent cell death. As ferroptosis can be influenced by various factors, there are many different molecular mechanisms involved in regulating cell ferroptosis as demonstrated in HCC. For example, the induction of ferroptosis in hepatocellular carcinoma cells, which has the effect of suppressing cell viability and proliferation, can be activated via the lnc-PVT1/miR-214-3p/GPX4 axis [8], TRIB2/*β*-TrCP/TFRC [9], or p62-Keap1-NRF2 pathways [10]. Thus, ferroptosis plays an important role in HCC carcinogenesis and can be used as a means for identifying potential gene therapy targets for use in the treatment of liver cancer. There are also many factors involved with the regulation of HCC. As important regulatory genes [11], long noncoding RNAs (lncRNAs) exert critical regulatory effects on a variety of physiological functions [12].

Among them, highly upregulated in liver cancer (HULC) is the most studied lncRNA related to liver cancer [13]. It has been reported that HULC mediates HCC cell death by regulating miR-186/HMGA2 [14], miR-134-5p/FOXM1 axis [15], miR-200a-3p/ZEB1 axis [16], and miR-377-5p/HIF-1*α* [17] as well as other signaling pathways. However, whether HULC is involved in the biological behavior of HCC cells as affected by ferroptosis remains unknown. In our study, we found that HULC knockdown induces ferroptosis in HCC cells. In this process, expressions of many miRNAs have been shown to be altered in HCC cells. Among these is miR-3200-5p, which plays an important role in the process of ferroptosis in HCC cells treated with the si-HULC. We have demonstrated that HULC can function as a competing endogenous RNA (ceRNA) of miR-3200-5p, enabling miR-3200-5p the ability to regulate the ferroptosis in HCC by targeting activating transcription factor 4 (ATF4). ATF4, a member of the CREB/ATF family [18], is the cAMP response element binding 2 (CREB2) [19] and can function as both a transcriptional activator and a repressor [20]. ATF4 can also serve as a protective gene for the endoplasmic reticulum [21] and oxidative [22] stress, thus regulating adaptative functioning of cells [23]. Our current results elucidate a novel mechanism indicating that downregulation of HULC induces ferroptosis in hepatocellular carcinoma by targeting the miR-3200-5p/ATF4 axis. Such findings offer the potential therapeutic targets of tumors based on lncRNA HULC.

## 2. Materials and Methods

### 2.1. Patient Specimens

Paraffin sections of HCC (*n* = 22) and normal liver (*n* = 17) tissues were obtained from the Jiaozuo People's Hospital over the period from 2016 to 2020. None of the patients received preoperative radiotherapy or chemotherapy prior to surgical resection. This study was approved by the Ethics Committee of the Jiaozuo People's Hospital, and all patients provided written informed consent for use of their tissue for research.

### 2.2. Cell Culture

Human HCC cell lines, SMMC-7721 and BEL-7402 (Fenghui Biotechnology Co., Ltd.), were cultured in an incubator (NEST Biotechnology Co., Ltd.). The nutrient solution consisted of a mixture of Dulbecco's modified Eagle's medium (DMEM) with 4.5 g/L glucose, L-glutamine (Corning, Inc., USA), 10% fetal bovine serum (FBS, Corning, Inc.), and 1% penicillin/streptomycin (Beijing Solarbio Science & Technology Co., Ltd.) with 95% air and 5% CO_2_ at 37°C.

### 2.3. Cell Transfection

SMMC-7721 and BEL-7402 cells were plated into 6- and 24-well plates and cultivated for 12-24 h. Subsequently, 50 nM of oligonucleotides and Lipofectamine 2000 (Invitrogen, Carlsbad, CA) was dripped into each well containing a monolayer of cells, according to the manufacturers' specifications. All oligonucleotides were purchased from RIBOBIO (Guangzhou, China) and included small-interfering RNA- (siRNA-) targeting HULC (si-HULC) and ATF4 (si-ATF4), siRNA negative control (si-NC), mimic miR-3200-5p and mimic NC, and inhibitor miR-3200-5p and inhibitor NC.

### 2.4. qRT-PCR

To obtain total RNA, HCC cells were lysed with the use of the Eastep® Super Total RNA Extraction Kit (Promega, Shanghai, China). To synthesize complementary DNA (cDNA), reverse transcription was immediately executed as achieved with the use of the NovoScript® Plus All-in-One 1st Strand cDNA Synthesis SuperMix (gDNA Purge) (Novoprotein Scientific Inc.; Shanghai, China) using 1 *μ*g RNA. The amplification reaction was then performed via the SYBR® Green qPCR Master Mix (Low ROX) on the ABI Prism® 7500 Real-Time PCR System (7500, ABI Company, USA) using the following conditions: 95°C for 5 min and 45 cycles of 95°C for 20 s and 60°C for 40 s. Relative expression levels were assessed using the 2^−*ΔΔ*Ct^ method, with the correction of glyceraldehyde-3-phosphate dehydrogenase (GAPDH, for HULC and ATF4) and small nuclear RNA U6 (for miR-3200-5p). All primer sequences consisted of the following: HULC (forward (F):5′-TCATGATGGAATTGGAGCCTT-3′; reverse (R):5′-CTCTTCCTGGCTTGCAGATTG-3′), ATF4 (F:5′ATGGATTTGAAGGAGTTCGACT-3′; R:5′-AGAGATCACAAGTGTCATCCAA-3′), and miR-3200-5p (F:5′GCGAATCTGAGAAGG-CGCA-3′; R:5′-AGTGCAGGGTCCGAGGTATT-3′).

### 2.5. MTT Assay

After replacement of 100 *μ*L fresh culture medium for the transfected SMMC-7721 and BEL-7402 cells in the 96-well plates, 10 *μ*L 3-(4,5-dimethylthiazol-2-y1)-2,5-diphenyl tetrazolium bromide (MTT; Solarbio) was pipetted into each well for 4 h, followed by the complement of 110 *μ*L formazan (Solarbio) to each well which was then shaken on a table blender for 10 min. The absorbance of the mixture was read by microplate reader (Gene Company Limited, BioTek, Epoch) at 490 nm. Relative cell viability (%) was calculated based on comparisons with control cells.

### 2.6. Luciferase Reporter Gene Assay

The miRWalk database was used to predict the binding site on the 5′-UTR of has-miR-3200-5p. The luciferase reporter gene assay was performed using the Dual-Luciferase Reporter Assay System (Promega, Madison, WI, United States) according to the manufacturer's instructions.

### 2.7. Colony Formation Assay

Transfected cells were seeded on 6-well plates with 600 cells per well. Then, transfected cells were maintained in DMEM1 (Corning, USA) containing 10% FBS, which was replaced every 3 days. Cells were then cultivated for 7 days in a humidified incubator with 5% CO_2_ at 37°C. Cells were then washed with PBS, fixed by methanol, and stained with crystal violet.

### 2.8. Western Blot

HCC cells were transfected for 48 h, the protein extracted on ice using cell lysis buffer, and the lysed cells collected. The obtained proteins were quantified using the BCA Protein Assay Kit (Solarbio). Proteins were separated using 10% SDS-PAGE and transferred to PVDF membranes. Membranes were blocked with 5% nonfat milk for 2 h and incubated overnight with anticaspase-3 (1 : 1000, Proteintech), Bcl-2 (1 : 1000, Proteintech), MMP-2 (1 : 1000, Proteintech), ATF-4 (1 : 1000, abcam), or GAPDH (1 : 2000, Proteintech), followed by incubation with HRP-conjugated antirabbit IgG H+L (1 : 3000, Proteintech) for 1 h at room temperature. Band intensities were measured using the Tanon 5200 Imaging System, and level analyses were performed using ImageJ.

### 2.9. Immunohistochemistry (IHC)

Paraffin sections (4 *μ*m) were cut and deparaffinized. Optimal staining was achieved with the heat-induced antigen retrieval method, using 10 mmol/L citric acid (pH 6.0). Slides were then incubated overnight with anti-ATF 4 antibodies (1 : 100, abcam) at 4°C. They were then rinsed with PBS and further incubated with an anti-IgG secondary antibody (1 : 200; Proteintech) at room temperature for 60 min, followed by diaminobenzidine staining.

### 2.10. Scratch Wound Healing Assay

When SMMC-7721 and BEL-7402 cells were cultured to a density of 50%, an artificial scratch was created using a 10 *μ*L pipette tip. Cell slides were then rinsed with PBS. Cells were cultured for 0, 24, or 48 h and observed under an inverted microscope (Olympus, Tokyo, Japan). The wound healing rate was calculated by the fraction of cell coverage across the scratch line.

### 2.11. Flow Cytometry

Assessment of cell death was performed using the Annexin V-PE/7-AAD Apoptosis Detection Kit (Keygen Biotech). Cells were detached by pancreatin without EDTA, with 5 × 10^5^ cells washed using PBS, resuspended in 50 *μ*L binding buffer, and stained with 1 *μ*L Annexin V-PE and 5 *μ*L 7-AAD. Cells were incubated for 15 min at 37°C in the dark followed by the addition of a 450 *μ*L binding buffer just prior to flow cytometry.

### 2.12. Determination of ROS Levels

Transfected cells were collected according to the instructions provided by the ROS Assay Kit (Beijing Solarbio Science &Technology Co., Ltd.) and cultured in 6-well plates incubated with a 10 *μ*mol/L DCFH-DA probe for 20 min at 37°C. To completely remove probes that did not enter cells, cells were washed three times in serum-free culture medium. Intracellular ROS levels of the samples were detected with the use of flow cytometry.

### 2.13. Determination MDA Generation

Transfected cells were collected according to directions of the MDA Assay Kit (Beijing Solarbio Science &Technology Co., Ltd.), and 1 mL of extract was added for every 5 × 10^6^ cells. Cells were fragmented 30 times with the use of an ultrasonic cell breaker, and crushed cells were centrifuged to obtain the supernatant. The samples obtained along with the working liquid were added to the 96-well plate according to the instructions, and absorbance was measured at 450, 532, and 600 nm on a microplate reader. Finally, the content of MDA was calculated according to the formula in the manual.

### 2.14. Determination GSH Levels

Transfected cells were collected according to instructions provided by the GSH Assay Kit (Beijing Solarbio Science &Technology Co., Ltd.). The standard solution with an initial the concentration of 1 mg/mL was diluted into concentrations of 300, 200, 100, 50, and 25 *μ*g/mL. A standard curve was then generated by measuring absorbance on a microplate reader at a wavelength 412 nm. Collected cell samples were added to the 96-well plates according to the instructions, and the absorbance was measured at a wavelength 412 nm on a microplate reader.

### 2.15. Determination Fe^2+^ Levels

Transfected cells were collected according to directions of the Iron Assay Kit (Dojindo Laboratories, All Rights Reserved) according to the manufacturer's instructions. The concentration of the iron divalent can be calculated by making a standard curve. The iron concentration of the original sample solution was calculated according to the dilution ratio of the sample.

### 2.16. Statistical Analysis

Statistical analysis was performed using Prism 8 (GraphPad) software. Data are shown as the means ± standard deviations (SD). An *F*-test was used to confirm whether a homogeneity of variance was present. For determinations of statisticaliy significant differences, one-way ANOVA was conducted followed by Dunnett's multiple comparison test for assessing pairwise differences. *p* value < 0.05 was required for results to be considered as statistically significant.

## 3. Results

### 3.1. Knockdown of HULC Decreases Viability and Proliferation of HCC Cells

**Figure 1:**
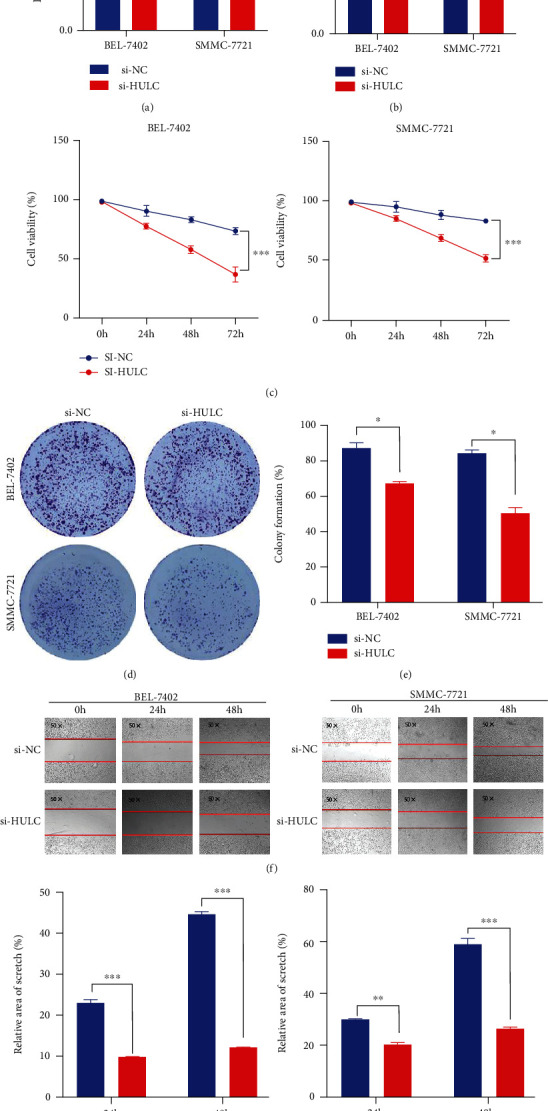
Knockdown of HULC decreases viability and proliferation of HCC cells. (a) The transfection efficiency of si-HULC was verified with use of qRT-PCR. (b) Iron detection in BEL-7402 and SMMC-7721 cells transfected with si-HULC. (c) MTT was used to analyze BEL-7402 and SMMC-7721 cell viabilities transfected with si-NC or si-HULC. (d, e) Colony formation assay was used to assess cell proliferation. (f, g) Representative images and quantification of BEL-7402 and SMMC-7721 cell migration following HULC depletion in a scratch wound healing assay. Three independent experiments and sample size (*n* = 3), compared with the si-NC; ^∗^*p* < 0.05; ^∗∗^*p* < 0.01; ^∗∗∗^*p* < 0.001.

Results obtained from the qRT-PCR assay revealed the downregulation of HULC in HCC cells by si-HULC transfection (Figure 1(a)). After HULC knockdown, the expression level of Fe^2+^ increased about 1.5 times compared with the negative control (Figure 1(b)). This effect was demonstrated in both BEL-7402 and SMMC-7721 cells treated with si-HULC for 72 h. Results obtained from the MTT assay supported these findings, as compared with those from the control group; HULC knockdown significantly inhibited the viability of the HCC cells in a time-dependent manner (Figure 1(c)). More specifically, we found that downregulation of HULC expression inhibited cell proliferation and colony formation in BEL-7402 and SMMC-7721 cells (Figures 1(d) and 1(e)). Wound healing assay results showed that downregulation of HULC decreased the migration rates within BEL-7402 and SMMC-7721 cells determined at 48 h. As shown in Figures 1(f) and 1(g), knockdown of HULC can significantly decrease the proliferation, invasion, and metastasis of HCC cells.

### 3.2. HCC Cell Death from HULC Downregulation Is Associated with Ferroptosis

**Figure 2:**
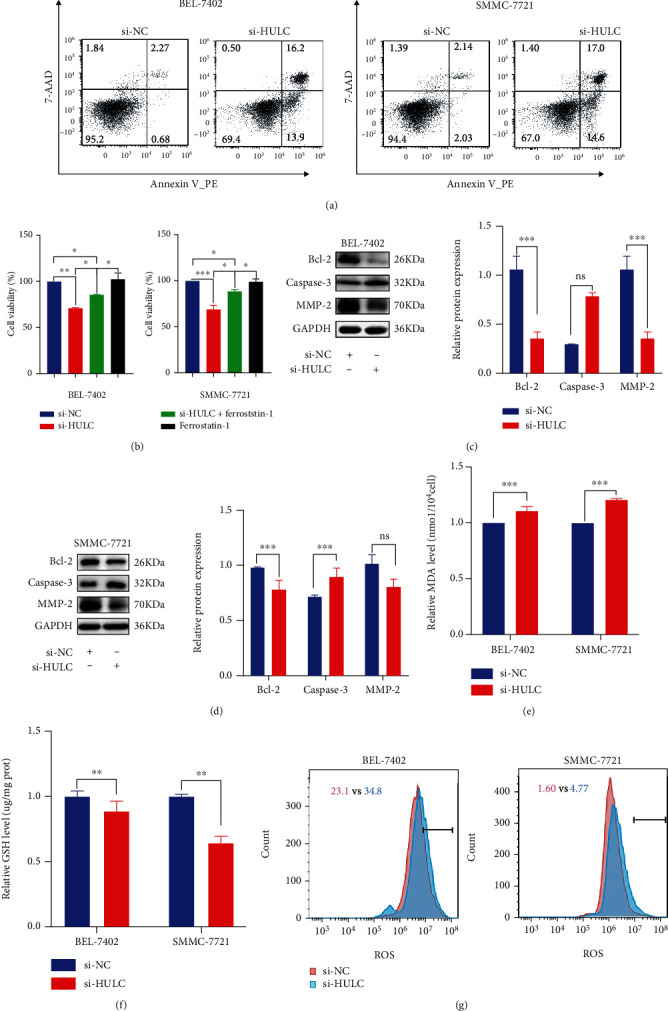
Downregulation of HULC induces ferroptosis. (a) Cell apoptosis was evaluated following transfection of si-HULC and si-NC by flow cytometry and western blotting analysis (c, d). (b) MTT assay was used to observe the activity of HCC cells treated with si-HULC in the presence of Ferrostatin-1. (e–g) MDA, GSH, and ROS were used for examining oxidative stress within cells after HULC knockdown. Three independent experiments and sample size (*n* = 3), compared with the control group; ^∗^*p* < 0.05; ^∗∗^*p* < 0.01; ^∗∗∗^*p* < 0.001.

Based on flow cytometry analysis, BEL-7402 and SMMC-7721 cells transfected with si-HULC showed an increase in apoptotic cells as compared with those transfected with si-NC (Figure 2(a)). To investigate whether Ferrostatin-1, an ferroptosis inhibitor, affects cell death after ferroptosis activation, si-HULC was used to observe the viability of HCC cells treated with si-HULC in the presence of Ferrostatin-1. The results showed that si-HULC treatment reduced cell viability compared with the negative control, but the concurrent addition of Ferrostatin-1 reversed HCC cell death; moreover, it was shown that the viability of HCC cells treated with si-HULC+Ferrostatin-1 was still fewer than that of the si-NC control (Figure 2(b)). As shown in Figures 2(c) and 2(d), protein expressions of Bcl-2 and MMP-2 were significantly decreased, while that of Caspase-3 significantly increased in the si-HULC-treated culture as compared with that of the control group. The ferroptosis-associated oxidative stress indicators MDA and ROS were significantly elevated, but the ferroptosis-suppressive indicator, GSH, was decreased in the si-HULC group (Figures 2(e)–2(g)).

### 3.3. HULC Targets miR-3200-5p and Functions as a ceRNA in HCC Cells

**Figure 3:**
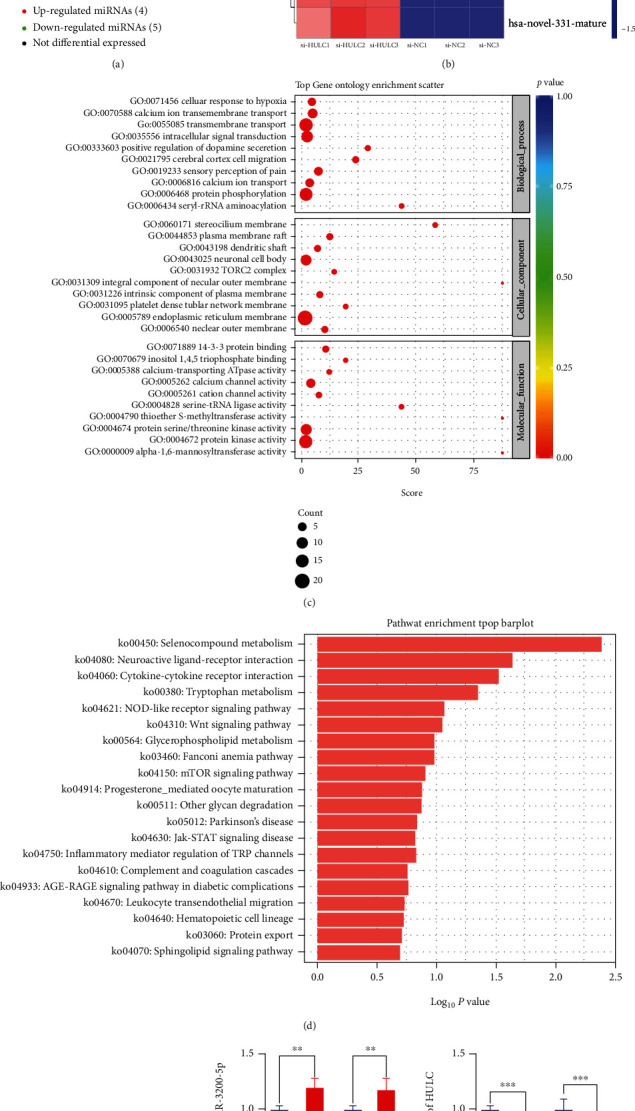
HULC targets miR-3200-5p and acts as a ceRNA. (a, b) Results of high-throughput sequencing identified a total of 9 significant differential genes. (c) Results of GO analysis revealed that target genes were related to selenocysteine lyase activity, peroxisomal mechanism targeting signal-1 binding, and tumor necrosis factor receptor superfamily binding. (d) KEGG pathway analysis demonstrated that selenium compound metabolism and neuroactive ligand receptor interaction along with the Wnt and JAK-STAT signaling pathways were the most abundant. (e) Results from the target prediction indicated that HULC might interact with miR-3200-5p. (f) qRT-PCR was performed to assess gene changes in miR-3200-5p after knockdown of HULC. (g) When the mimic miR-3200-5p was added, a decrease in HULC expression was observed in BEL-7402 and SMMC-7721 cells. Three independent experiments and sample size (*n* = 3), compared with the control group; ^∗∗^*p* < 0.01; ^∗∗∗^*p* < 0.001.

After HULC knockdown, results of high-throughput sequencing revealed nine significantly different miRNAs, among which four miRNAs were upregulated and the rest of miRNAs were downregulated (Figures 3(a) and 3(b)). GO and KEGG pathways were then analyzed. Target genes of the upregulated miRNAs were related to selenocysteine lyase activity, peroxisomal mechanism targeting signal binding, peroxisomal targeting sequence binding, and tumor necrosis factor receptor superfamily binding (Figure 3(c)). KEGG pathway analysis showed that selenium compound metabolism and neuroactive ligand receptor interaction, along with the Wnt and JAK-STAT signaling pathways, were the most abundant and relevant pathways identified (Figure 3(d)). Results from bioinformatics analysis, as achieved with the use of the bioinformatics prediction website, indicated that HULC might interact with miR-3200-5p (Figure 3(e)). qRT-PCR was then performed to determine whether expressions of miR-3200-5p and HULC were presented in BEL-7402 and SMMC-7721 cells after relevant transfection. When transfected with the si-NC, si-HULC, there was a decrease in the expression of miR-3200-5p (Figure 3(f)), and the miR-3200-5p mimic decreased the HULC expression in HCC cells (Figure 3(g)). Based on these results, we conclude that a ceRNA component exists between HULC and miR-3200-5p.

### 3.4. Modification of miR-3200-5p Levels in HCC Cells

**Figure 4:**
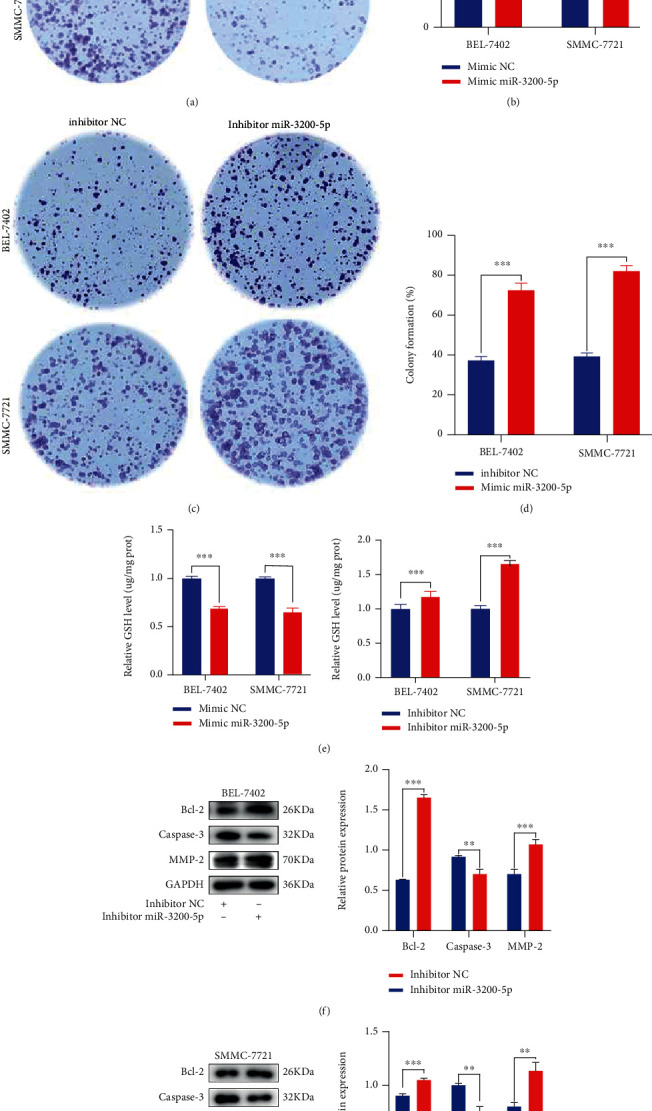
Modification of miR-3200-5p levels in HCC cells. (a–d) Cell colonies were used to assess the proliferation of cells in response to the miR-3200-5p mimic/inhibitor. (e) GSH levels were analyzed in BEL-7402 and SMMC-7721 cells transfected with the miR-3200-5p mimic/inhibitor. (f, g) Western blotting analysis was used for estimating the effects of the miR-3200-5p inhibitor or inhibitor NC on protein levels. Three independent experiments and sample size (*n* = 3), compared with the control group; ^∗∗^*p* < 0.01; ^∗∗∗^*p* < 0.001.

Cell colonies were subsequently established to detect the proliferation of the transfected cells by the mimic and inhibitor of miR-3200-5p. These result indicated that an overexpression of miR-3200-5p inhibits the HCC cell clonal formation (Figures 4(a) and 4(b)), while suppression of miR-3200-5p promoted the HCC cell clone formation ability (Figures 4(c) and 4(d)). As shown in Figure 4(e), results from GSH assays revealed that a promotion in the expression of miR-3200-5p produced a significant decrease in antioxidative indicators, while inhibiting miR-3200-5p expression increased antioxidative indicators. In addition, inhibiting miR-3200-5p promotes the expressions of Bcl-2/MMP-2 and inhibits Caspase-3 expression as compared with the control group (Figures 4(f) and 4(g)).

### 3.5. lncRNA HULC Mediates Ferroptosis through miR-3200-5p

**Figure 5:**
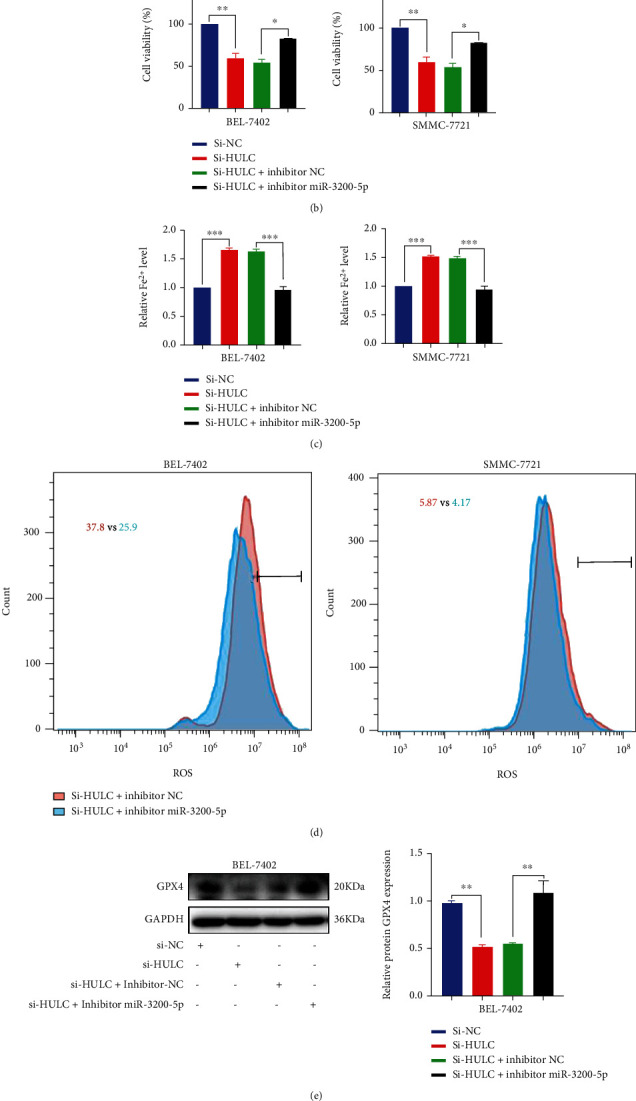
lncRNA HULC mediates ferroptosis through miR-3200-5p. (a, b) BEL-7402 and SMMC-7721 cells were cotransfected with si-HULC and a miR-3200-5p mimic/inhibitor; MTT levels were used to assess cell viability. (c, d) si-HULC and inhibitor miR-3200-5p were used to detect the production of Fe^2+^ and ROS, reflecting the ferroptosis of cells. (e, f) Western blot was used to detect the expression of ferroptosis-related protein GPX4 in hepatocellular carcinoma cells. Three independent experiments and sample size (*n* = 3), compared with the control group; ^∗^*p* < 0.05; ^∗∗^*p* < 0.01; ^∗∗∗^*p* < 0.001.

The bidirectional relationship between HULC and miR-3200-5p was enhanced by inducing HULC and mimicking miR-3200-5p cell viability. MTT assay results demonstrated that HCC cell viability decreased after addition of the miR-3200-5p mimic. When combined with si-HULC, cell viability decreased more significantly (Figure 5(a)). The findings indicate that miR-3200-5p can induce oxidative stress in hepatoma cells and raises the issue of whether miR-3200-5p-induced oxidative stress can affect ferroptosis. MTT assay results demonstrated that si-HULC can inhibit the expression of HULC to decrease HCC cell viability; however, when pretreated with the miR-3200-5p inhibitor, cell viability is increased in HCC cells (Figure 5(b)). The capacity for the miR-3200-5p inhibitor to reverse cell viability was significantly reduced following HULC knockdown in BEL-7402 and SMMC-7721 cells. Meanwhile, Fe^2+^ and ROS production was detected in BEL-7402 and SMMC-7721 cells; when HULC was silenced, iron expression increased, while adding an inhibitor partially reversed iron divalence and oxidative stress levels (Figures 5(c) and 5(d)). Western blot showed that the expression of ferroptosis-related protein GPX4 decreased after HULC knockdown, and the GPX4 expression level was reversed when the inhibitor miR-3200-5p was added simultaneously (Figures 5(e) and 5(f)). Taken together, these findings suggest that miR-3200-5p inhibits tumor progression and enhances ferroptosis in HCC cells via inhibiting HULC.

### 3.6. miR-3200-5p Targets ATF4 in HCC Cells

**Figure 6:**
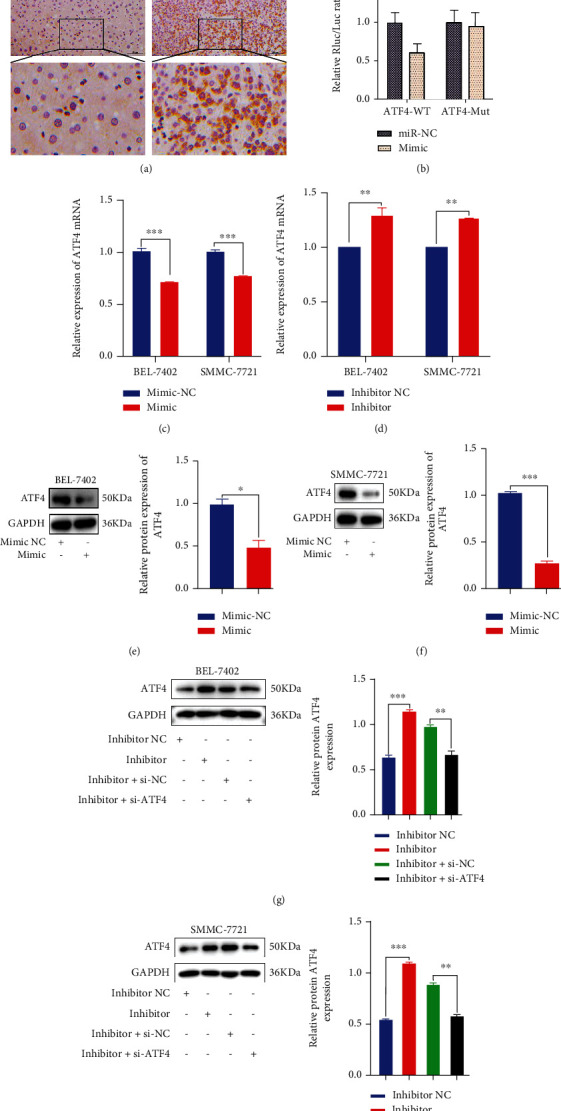
miR-3200-5p targeted ATF4. (a) The expression of ATF4 in HCC (*n* = 22) and normal liver (*n* = 17) tissues was detected with the use of immunohistochemistry. (b) The results of the double luciferase reporter gene showed that the gene ATF4 was associated with miR-3200-5p. (c, d) qRT-PCR was performed to detect the expression of ATF4 mRNA in HCC cells transfected with miR-3200-5p mimic/inhibitor. (e–h) Western blot was performed to detect ATF4 expression following the addition of the mimic miR-3200-5p, inhibitor miR-3200-5p, inhibitor miR-3200-5p+si-NC, or inhibitor miR-3200-5p+si-ATF4 in BEL-7402 and SMMC-7721 cells. Three independent experiments and sample size (*n* = 3), compared with the control group; ^∗^*p* < 0.05; ^∗∗^*p* < 0.01; ^∗∗∗^*p* < 0.001.

Based on predictions from bioinformatics data, ATF4, which is mainly expressed in the cytoplasm with only small amounts in the nucleus, may be a potential target of miR-3200-5p. As shown in Figure 6(a), high expression levels of ATF4 were found in HCC tissue as compared with matched normal liver controls. Results of the double luciferase reporter gene assay showed that the luc ratio of ATF4-WT and ATF4-MUT decreased after the addition of the miR-3200-5p mimic (Figure 6(b)). In hepatocellular carcinoma cells, results from experiments involved with transient transfections of miR-3200-5p suggested that miR-3200-5p could directly bind to ATF4. In contrast, the miR-3200-5p mimic significantly reduced ATF4 mRNA, as revealed in qRT-PCR assays (Figure 6(c)). In BEL-7402 and SMMC-7721 cells, the promotion of miR-3200-5p inhibited the expression of ATF4 (Figures 6(e) and 6(f)), while suppression of miR-3200-5p enhanced the expression of ATF4. When BEL-7402 and SMMC-7721 cells were cotransfected with the ATF4 siRNA and miR-3200-5p inhibitor, there was a decrease in the protein expression of ATF4 (Figures 6(g) and 6(h)). These findings suggest that a targeted regulatory association exists between ATF4 and miR-3200-5p.

### 3.7. miR-3200-5p Regulates Ferroptosis in HCC via Targeting ATF4

**Figure 7:**
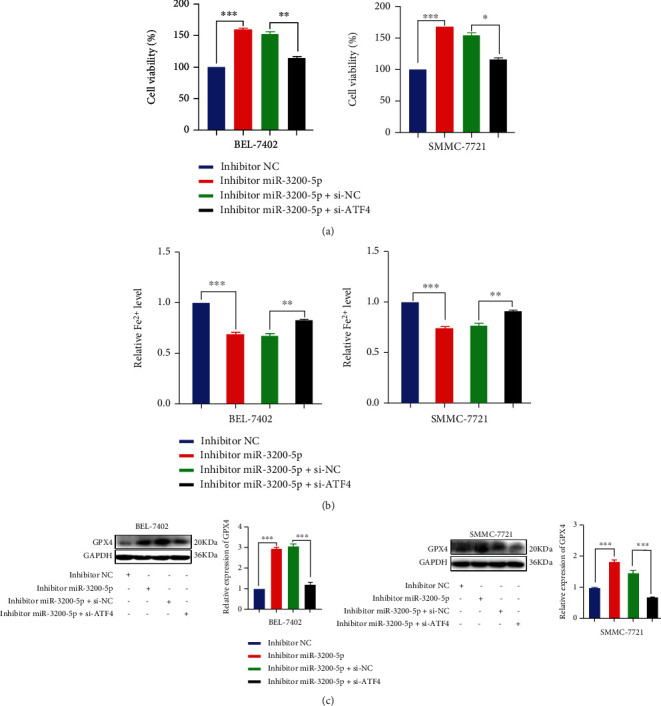
miR-3200-5p regulates ferroptosis via targeting ATF4. (a) Results of the MTT assay revealed that ATF4 siRNA transfection restored the capacity for promotion of proliferation as produced by the miR-3200-5p inhibitor. (b) Iron detection assay was used to detect ferroptosis after adding inhibitor miR-3200-5p and si-ATF4 into HCC cells. (c) Western blot was used to detect the effects of inhibitor miR-3200-5p and si-ATF4 on the levels of ferroptosis-related protein GPX4 in HCC cells. Three independent experiments and sample size (*n* = 3), compared with the control group; ^∗^*p* < 0.05; ^∗∗^*p* < 0.01; ^∗∗∗^*p* < 0.001.

The studies described above clearly indicate that miR-3200-5p targets ATF4 in HCC; however, whether the induced cell death involves ferroptosis remains unknown. In BEL-7402 and SMMC-7721 cells, ATF4 siRNA transfection reduced the proliferation capacity produced by the miR-3200-5p inhibitor and increased Fe^2+^ levels (Figures 7(a) and 7(b)). Western blot results showed that the expression level of ferroptosis-related protein GPX4 increased after the addition of the inhibitor miR-3200-5p in HCC cells, while the expression level of GPX4 was reversed after the addition of si-ATF4 (Figure 7(c)). Therefore, we come to a conclusion that miR-3200-5p regulates ferroptosis in HCC via targeting ATF4.

## 4. Discussion

In the present study, knockdown of long noncoding RNA HULC inhibits the malignant biological behavior of hepatocellular carcinoma cells. Recent evidence shows that HULC upregulates CyclinD1 through the miR-675-PKM2 pathway, promotes the growth of human hepatoma cells through autophagy [24], and accelerates liver cancer by inhibiting PTEN via autophagy cooperation with miR-15a [25]. The results of these studies suggest that an inhibition of HULC and interference of miR-107 targets Atg12 to promote invasion and metastasis of HCC [26]. Our current results extend these findings and further demonstrate that knockdown of HULC induces ferroptosis in HCC cells, and Ferrostatin-1 treatment partially reversed HCC cell death. Our experimental results are similar to previous reports [27, 28]

Ferroptosis represents a recently identified type of regulated, iron-dependent cell death [29] characterized by ferrous iron accumulation and lipid peroxidation [30]. A growing body of evidence has demonstrated that ferroptosis terminates and then ruptured the outer mitochondrial membrane producing dysfunction and toxic lipid peroxidation [31]. Ferroptosis plays an important role in inhibiting cancer growth and proliferation [32] which, in part, results from an excessive accumulation of ROS and an accentuation of the phenotype as accomplished by the filling of glutathione and consumption iron in cells [33]. It is genetically, biochemically, and morphologically distinct from other types of programmed cell death, such as apoptosis, autophagy, and necrosis [34]. However, HULC can exert effects upon ferroptosis through altering these functions via imparting changes to the miRNA. HULC has been identified as a ceRNA of miR-512 that promotes contrast-induced nephropathy (CIN) [35]. Meanwhile, miR-622 can target HULC to suppress the invasion and migration of human pancreatic cancer cells [36]. HULC knockdown has been shown to reverse LPS-induced sepsis via the regulation of miR-128-3p/RAC1 in HMEC-1 cells [37]. Accordingly, results as generated from these studies indicate that HULC affects miRNA by a ceRNA mechanism. The changes in miRNAs observed by high-throughput sequencing suggest that miR-3200-5p is the most critical. This regulatory mechanism of miR-3200-5p is competitively inhibited by DARS-AS1 and targets the cytoskeleton-associated protein 2 (CKAP2) to aggravate the growth and invasion of hepatocellular carcinoma [38]. In addition, miR-3200-5p acts as a sponge for circMTO1 [39] and LINC00324 [40] to attenuate tumorigenesis of gastric carcinoma and promotes osteosarcoma cell migration and invasion by negatively regulating BRMS1 expression [41]. Taken together, the studies summarized above clearly indicate that miR-3200-5p can induce autophagy in some cancer cells.

With HULC knockdown, expression levels of miR-3200-5p are clearly increased, and these increases in the miR-3200-5p expression are associated with increases in ferroptosis. Therefore, our findings demonstrate that HULC can affect ferroptosis in HCC cells by regulating miR-3200-5p. In general, miR-3200-5p functions by influencing its target gene, activating transcription factor 4 (ATF4). Through bioinformatics analysis, we were able to identify a miR-3200-5p mimic, which, when added to BEL-7402 and SMMC-7721 cells, decreased expression levels of ATF4. In contrast, expression levels of ATF4 increased after the addition of an miR-3200-5p inhibitor. These results demonstrate that a targeted regulatory relationship exists between miR-3200-5p and ATF4. ATF4 expression is upregulated in a variety of tumors [42] and plays a role in the response to hypoxia and amino acid deficiencies as well as endoplasmic reticulum [43] and oxidative [20, 44] stress. The effects of ATF4 on oxidative stress may also affect ferroptosis within HCC cells; our current results suggest that ATF4 affects antioxidant or prooxidation indices to influence HCC ferroptosis.

In conclusion, our results reveal that knockdown of HULC affects ferroptosis and changes in the biological behavior of hepatoma cells. Further, we found that HULC mediates ferroptosis within HCC cells by targeting ATF4 through miR-3200-5p. These findings demonstrating that HULC affects the ferroptosis of HCC cells through miR-3200-5p/ATF4 provide a crucial foundation for the development of new therapeutic targets in the prevention and treatment of liver cancer.

## Data Availability

All data generated or analyzed during this study are available in this article.
